# Effect of *D. melanogaster* larval density on pupal size

**DOI:** 10.17912/micropub.biology.000959

**Published:** 2023-12-04

**Authors:** Morgan Magee, Rebecca Spokony

**Affiliations:** 1 Macaulay Honors College, CUNY, New York, NY, United States; 2 Queens College, CUNY, New York, New York, United States; 3 Baruch College, CUNY, New York, New York, United States; 4 The Graduate Center, CUNY, New York, New York, United States

## Abstract

Many genetic pathways and environmental factors have been shown to affect *Drosophila melanogaster* adult body size. Larval density often varies considerably between vials, even when the same number of females of the same genotype are allowed to lay eggs in the vials for the same amount of time. To more accurately quantify the effects that larval population density has on pupal size, we established cultures of 1, 2, 10, 25, 50, 75 or 100 first instar larvae into vials and measured pupal length. We collected Oregon-R eggs on apple juice plates in six different cages and generated replicate cultures. We found that pupal size decreases as larval density in the culture increases by 25 individuals. The difference between male and female length remained relatively constant at each density (0.2 mm), but overall size decreased. The mean size differences between vials with 1 larvae and 100 larvae is 0.1(+/-0.02) mm in females and 0.11(+/-0.02) mm in males. These results suggest that fecundity and sex ratio could complicate results in Drosophila size studies.

**Figure 1. Pupal size decreases with small changes in vial density:**
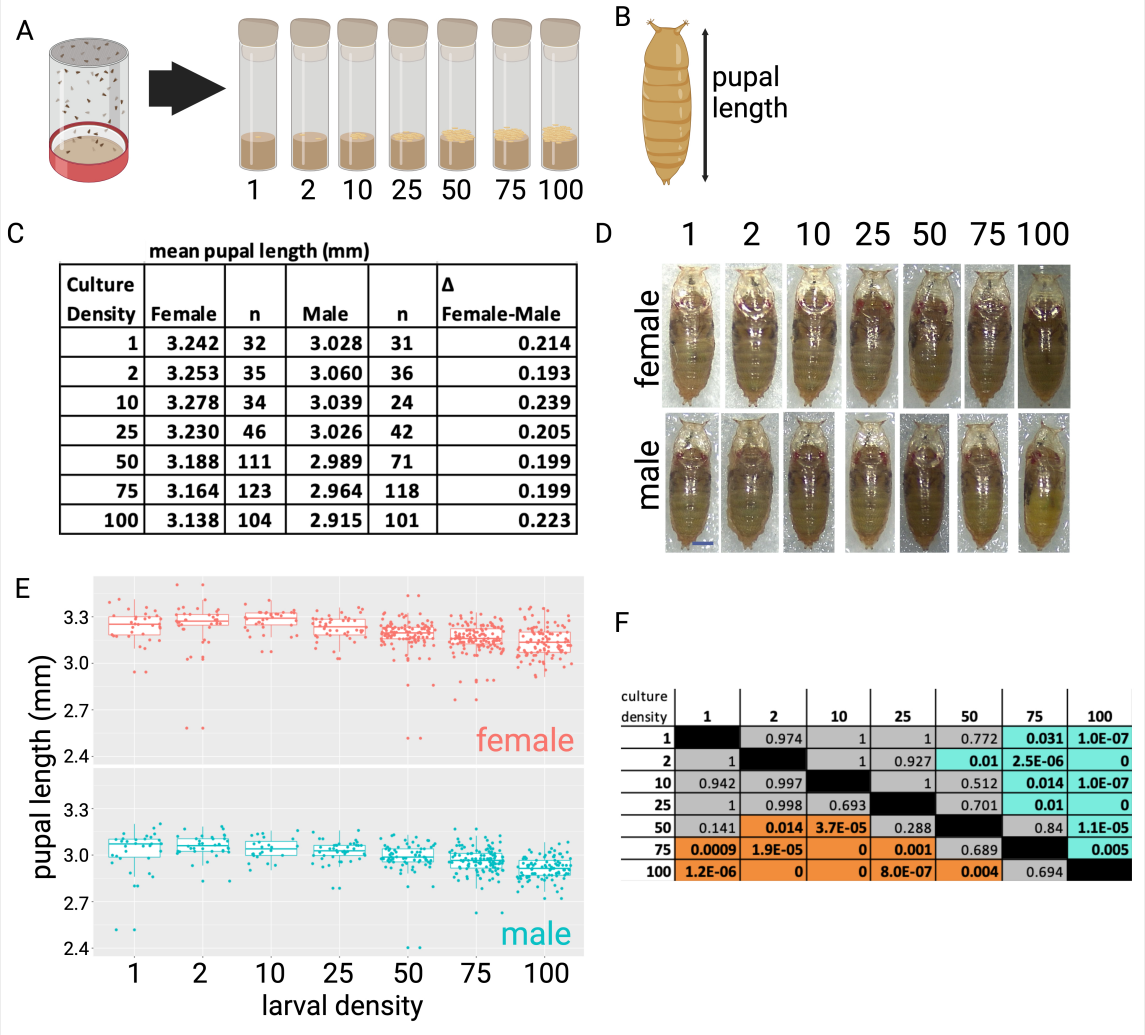
Pupal size decreases as larval density increases in the culture vials (larvae per vial: 1, 2, 10, 25, 50, 75, or 100). All assays were performed at 25º C. (A) Six replicate population cages contained adults while they laid eggs overnight on apple juice plates. First instar larvae were collected from the plates and transferred to individual vials. (B) Pupal length was measured from the top to the button of the pupal case, excluding the trachea. (C) Pupal length decreases as vial density increases. Males are smaller than females at all culture densities examined (p<0.01). The size difference between the males and the females was not statistically significantly different when compared between the different culture densities. (D) Example male and female pupae that are approximately the mean length for each culture density. Scale bar 0.5 mm (E) Box-plot of all data, each point is one sample. (F) Table of p-values from an ANOVA comparing all densities to one another. Female data is to the left, below the diagonal; comparisons where p<0.05 are highlighted in orange. Male data is to the right, above the diagonal; comparisons where p<0.05 are highlighted in blue.

## Description

Body size is coordinated through a wide array of environmental and genetic factors. Although it is known that increasing density of larvae leads to smaller adult body size, most of these studies have been conducted using high density vials (100s of larvae per vial, Bitner-Mathe 1999). Adult thorax size is affected by a difference of 90 larvae (Bitner-Mathe & Klaczko, 1999) and wing length is decreased by an increase of 30 larvae (Santos et al 1993) . Previous studies examining the effects of small changes (20) in larval culture density on pupal size pooled measurements of males and females despite the fact that body size differs significantly between the sexes (Reeves and Tautz 2017) .

Many studies of Drosophila use relatively small numbers of flies per vial, 8 females, laying eggs overnight (Greenspan, 2004). Adult females lay approximately 50 eggs per day (Bouletreau, 1978), leading to approximately 400 eggs per vial. Despite this, there is often significant amounts of variation in the amount of larvae per vial, making it important to know if density differences even in low density vials are enough to change adult size.

In this work we investigated whether the amount of variation in larval density often found in vials with the same number of female parents is enough to cause a difference in physiological characteristics. We collected eggs on apple juice plates and transferred 1st instar larvae to vials where the food had been chopped up to make it easier for the larvae to eat. We collected pupae and measured their lengths and widths.

We found that pupal length is inversely proportional to larval density, with relatively small increases in the number of larvae per vial leading to significant decreases in the length of both males and females. Pupal size decreases 3.2% (SD +/-0.1 mm) in females and 3.9% (SD +/-0.1 mm) in males between vials with 1 larvae and vials with 100 larvae. Despite male size appearing to decrease more than female size, this difference was not statistically significant.

Overall, these results illustrate that *D. melanogaster* larvae are very sensitive to vial density. In order to detect small effects on size, it is important to control for the number of larvae in each vial.

## Methods

Genetics and Husbandry

Oregon-R (OreR) flies were obtained from the Bloomington Drosophila Stock Center (BDSC stock number 25211). All stages of the experiments were maintained at 25 ℃.

Population cages containing 40-50 adult females and 10 adult males were sealed with agar apple juice plates to encourage the flies to lay eggs. Cages were made of disposable plastic beakers with air holes poked on the bottom (Fisher Scientific Cat# 14-955-1111B). The apple juice plates contain 75% water, 25% apple juice (Mott’s 100% juice), 2.5% sucrose (ShopRite Extra Fine Granulated Sugar) and 2.25% agarose (Apex BioResearch Products Cat# 20-101) solidified onto a 60 mm diameter petri dish (Genesee scientific cat #32-105G). A drop of yeast paste made by mixing water with enough dry yeast until it was a spreadable consistency was applied to the center of each plate before attaching it to each cage.

First instar larvae were individually transferred to wide Drosophila polystyrene vials (28.5 mm) with 12 mL of molasses food from Arcon Scientific (catalog #B20301). The BDSC molasses food recipe is used by Arcon, 86% water, 0.574% agar, 6.3% cornmeal, 1.52% yeast, 4.65% dry molasses, 0.39% propionic acid, 0.15% methylparaben, 0.52% ethanol. Food in each vial was crushed and mixed prior to inserting the larvae directly onto the food in the vials. Pupae were later collected from the sides of the vial and placed in dishes with damp filter paper to be measured.

Measurements

Pupae were photographed on filter paper at 80X magnification with Leica EZ4 microscopes and Leica LAS EZ software. A ruler was used to determine the scale, 1250 pixels for 10 mm. Measurements were taken from the top of the pupae to the bottom of the pupae using the line tool in ImageJ (Schneider et al., 2012) . RStudio (R Core Team, 2013; RStudio Team, 2020) was used to graph data and run ANOVA to determine if the differences in mean length at various densities are significantly different.

## Reagents

Drosophila genotype: OreR
